# Kisspeptin and RFRP-3 differentially regulate food intake and metabolic neuropeptides in the female desert jerboa

**DOI:** 10.1038/srep36057

**Published:** 2016-11-02

**Authors:** Rajae Talbi, Marie-Pierre Laran-Chich, Rabia Magoul, Seloua El Ouezzani, Valérie Simonneaux

**Affiliations:** 1 Laboratory of Neuroendocrinology and Nutritional and Climatic Environment, Faculty of Sciences, University Sidi Mohammed Ben Abdellah, BP 1796-ATLAS, FES, Morocco; 2 Institut des Neurosciences Cellulaires et Intégratives, UPR CNRS 3212, Université de Strasbourg, 5 rue Blaise Pascal, 67084 Strasbourg, France

## Abstract

Jerboas are wild rodents exhibiting exceptional adaptation to their desert environment. Under harsh autumn conditions, they shut down reproduction, increase body weight and hibernate, while during spring they become sexually active even under negative energy-balance. We recently reported that these rhythms are associated with synchronized changes in genes expressing reproductive (*Kiss1*, *Rfrp*) and metabolic (*Npy* and *Pomc*) peptides, raising the hypothesis of coordinated seasonal regulation of both functions. Here we analyzed whether kisspeptin and RFRP-3 regulate food-intake in parallel to their established reproductive functions. Intracerebroventricular administration of kisspeptin inhibited food intake by 1.5-fold in fasted, but not *ad-libitum* fed, female jerboas captured in spring, an effect associated with an increase in *Pomc* and decrease in *Rfrp* mRNA levels. By contrast, intracerebroventricular injection of RFRP-3 induced a 4-fold increase in food-intake in *ad-libitum* female jerboas, together with a decrease in *Pomc* and increase in *Npy* mRNA levels. This orexigenic effect of RFRP-3 was observed in both spring and autumn, whereas kisspeptin’s anorexigenic effect was only observed in spring. Altogether, this study reports opposite metabolic effects of kisspeptin and RFRP-3 in the female jerboa and strengthens our hypothesis of a coordinated, season-dependent, regulation of reproductive activity and food intake through interactions of these hypothalamic peptides.

An increasing number of studies support that the regulation of reproduction is intimately linked to energy homeostasis, especially in wild species exposed to marked seasonal changes in environmental light, temperature and food availability. Major progress has been made recently in the understanding of central mechanisms governing reproductive activity with the finding that two hypothalamic peptides, kisspeptin (Kp) and RF amide-related peptide 3 (RFRP-3, also known as gonadotropin inhibitory hormone) regulate GnRH neuronal activity and gonadotropin secretion^1^,^2^. In this study, we examine whether these two reproductive neuropeptides alter metabolism by investigating their effect on food intake and the metabolic neuropeptides proopiomelanocortin (POMC) and neuropeptide Y (NPY) in a wild hibernating seasonal rodent captured from its natural biotope, the jerboa (*Jaculus orientalis*).

Kp and RFRP-3, expressed in neurons localized either in the arcuate (ARC) and anteroventral periventricular (AVPV) nuclei or the medial hypothalamus, respectively, are now recognized to play pivotal roles in the central control of reproduction. Since the milestone finding that mutations in the gene encoding the Kp receptor result in disruption of reproductive function in both humans and mice^3^,^4^, this neuropeptide has been reported to potently activate GnRH release and the downstream pituitary-gonadal axis in a large number of mammals including rodents, sheep, primates and humans^5^,^6^,^7^. RFRP-3 was first shown to inhibit GnRH neuronal activity and gonadotropin secretion in mammals^8^,^9^,^10^,^11^,^12^ until two recent studies reported that it can also activate the gonadotropic axis in male Syrian and Siberian hamsters^13^,^14^. Notably, Kp and RFRP-3 content display marked photoperiodic variation in seasonal species^15^,^16^,^17^,^18^,^19^ suggesting a pivotal role of both neuropeptides in the synchronization of reproductive activity with the seasons^20^.

Furthermore, several studies indicate that both peptides may also alter food intake. RFRP-3 increases food intake in rodents, sheep and non-human primates^21^,^22^,^23^,^24^ while Kp displays an anorexigenic effect in overnight fasted mice^25^. In line with these feeding behaviors, other studies indicate that both peptides may regulate POMC or NPY arcuate neurons. POMC is an anorexigenic precursor polypeptide that inhibits food intake, a process mainly mediated by α-MSH^26^. NPY on the other hand displays a strong orexigenic effect^27^. In sheep, Kp fibers are in close apposition to POMC and NPY neurons and Kp treatment decreases POMC and increases NPY gene expression^28^, while in mice, Kp activates POMC and inhibits NPY neurons^29^. On the other hand, RFRP-3 projects to NPY and POMC neurons in the ewe^30^, attenuates the action of Kp on POMC neurons in mice^29^ and increases NPY gene expression in the rat^23^.

We recently reported a unique coordinated springtime activation of genes encoding peptides involved in reproduction (*Kiss1* and *Rfrp*) and metabolism (*POMC* and *somatostatin*) in the wild jerboa^31^. The jerboa is a particularly interesting animal model living in a semi-desert milieu characterized by large annual variations in environmental temperature, water and food supply. In autumn/winter, when the natural conditions are unfavorable, jerboas shut down their reproductive activity^32^,^33^, increase their food intake and body weight and hibernate^34^,^35^, while in spring/summer when favorable conditions return, the animals display opposite regulations with a reactivation of reproduction and reduction in body weight and food intake^32^,^33^,^35^,^36^.

Altogether, these observations suggest that the jerboa’s reproduction and energy homeostasis may be strictly coordinated in order to ensure optimal synchronization between reproduction and offspring survival in a marked seasonal environment. Therefore, the aim of this study was to assess whether the reproductive neuropeptides Kp and RFRP-3 regulate food intake and metabolic neuropeptides, taking into account putative sex differences and seasonal effects.

## Results

### Effect of central injection of Kp10 on food intake and hypothalamic peptide gene expression in fasted female and male jerboa in spring or autumn

**Figure 1:**
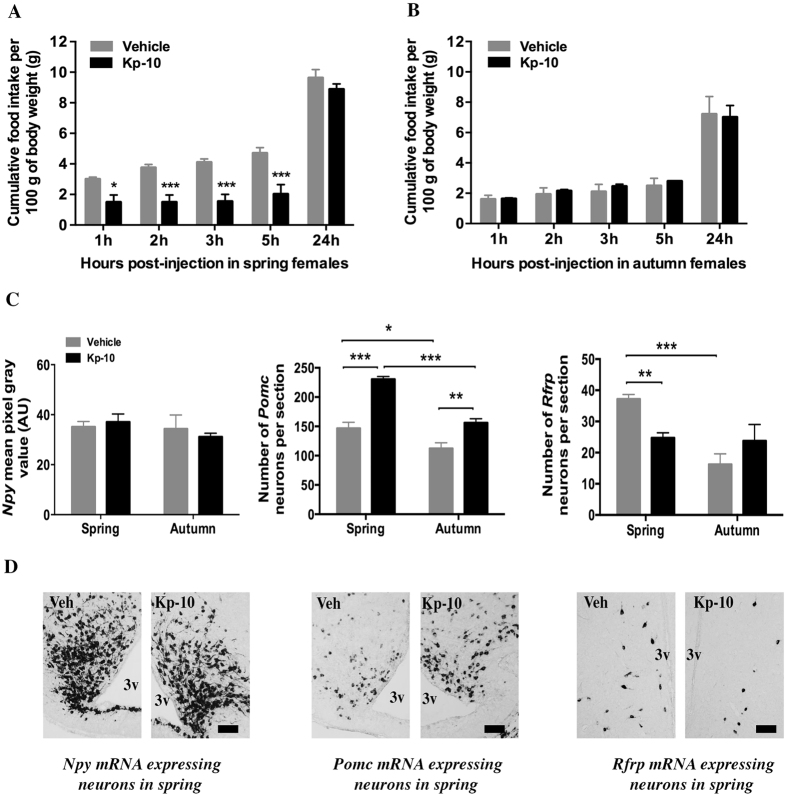
Effect of acute central injection of Kp10 on food intake and expression of genes encoding hypothalamic peptides NPY, POMC and RFRP-3 in 48 hours fasted female jerboas at spring and autumn. (**A**,**B**) Cumulative food intake (g food intake/100 g body weight) measured 1 h, 2 h, 3 h, 5 h, and 24 h following icv injection of 4 μg Kp10 or vehicle (0.9% NaCl) in 48 h-food deprived female jerboas captured in spring (**A**) or autumn (**B**). Statistical evaluation was performed using two-way repeated measures ANOVA followed by Student-Newman-Keuls test; data are expressed as mean ± SEM (n = 4 per group). ***p < 0.001 and *p < 0.05 for significant differences between Kp10 and vehicle injected groups. (**C**) Semi-quantitative analysis of the labeling intensity of *Npy* mRNA in the arcuate nucleus (upper left panel), and quantitative measure of the number of neurons expressing *Pomc* mRNA in the arcuate nucleus (upper middle panel) and *Rfrp* mRNA in the dorso/ventro medial hypothalamus (upper right panel) 1h30 after 4 μg Kp10 (black bars) or vehicle (grey bars) icv injections in spring and autumn female jerboas. Statistical evaluation was performed using two-way ANOVA followed by a post hoc Holm-Sidak test; data are presented as mean ± SEM (n = 4 in the spring group; n = 3 in the autumn group). ***p < 0.001 and **p < 0.01 for significant differences between Kp10 and vehicle injected groups and ***p < 0.001 and *p < 0.01 for differences between spring and autumn groups. (**D**) Representative images of *Npy* mRNA labeling in the arcuate nucleus (lower left panel), *Pomc* mRNA labeling in the arcuate nucleus (lower middle panel) and *Rfrp* mRNA labeling in the DMH/VMH (lower right panel) of jerboas sacrificed in spring 1h30 after Kp10 or vehicle icv injections, Scale bars represent 100 μm. AU: arbitrary units, 3v; third ventricle; Veh: vehicle.

**Figure 2:**
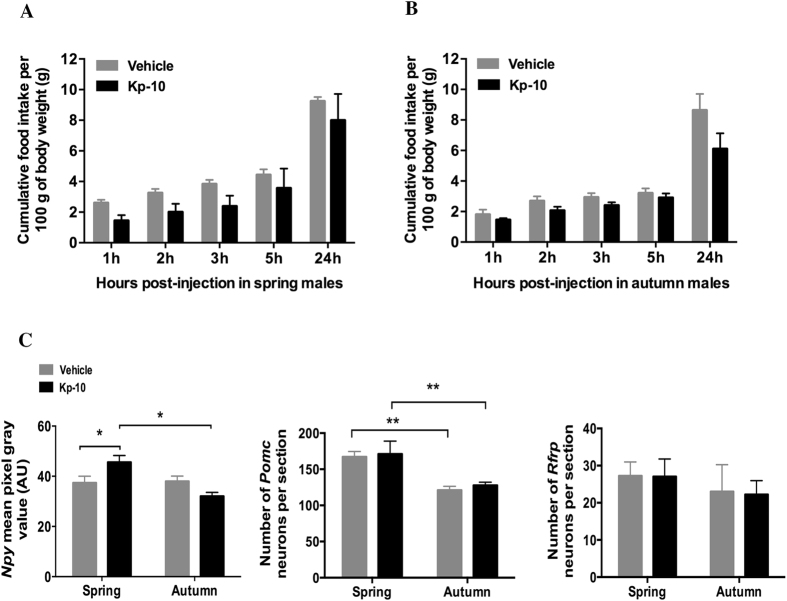
Effect of acute central injection of Kp10 on food intake and expression of genes encoding hypothalamic peptides NPY, POMC and RFRP-3, in 48 hours fasted male jerboas at spring and autumn. (**A,B**) Cumulative food intake (g food intake/100 g body weight) measured 1 h, 2 h, 3 h, 5 h, and 24 h following icv injection of 4 μg Kp10 or vehicle (NaCl 0.9%) in 48 h-food deprived male jerboas captured in spring (**A**) or autumn (**B**). Statistical evaluation was performed using two-way repeated measures ANOVA followed by Student-Newman-Keuls test; data are expressed as mean ± SEM (n = 4 per group). No significant difference was observed between Kp10 and vehicle injected jerboas. (**C**) Semi-quantitative analysis of the labeling intensity of *Npy* mRNA in the arcuate nucleus (upper left panel), and quantitative measure of the number of neurons expressing *Pomc* mRNA in the arcuate nucleus (upper middle panel) and *Rfrp* mRNA in the dorso/ventro medial hypothalamus (upper right panel) 1h30 after 4 μg Kp10 (black bars) or vehicle (grey bars) icv injections in spring and autumn female jerboas. Statistical evaluation was performed using two-way ANOVA followed by a post hoc Holm-Sidak test; data are presented as mean ± SEM (n = 4 per group). *p < 0.05 for significant differences between Kp10 and vehicle injected group and **p < 0.01 for differences between spring and autumn groups. A.U: arbitrary unit.

Although a single dose of 4 μg Kp10 was enough to increase by 5 fold the circulating testosterone in male jerboas (see material and methods section), the peptide displayed no effect on food intake when compared to control *ad-libitum* fed male and female jerboas (data not shown). Based on published data where Kp10 inhibited food intake in fasted mice^25^, the effect of the peptide was next evaluated on 48 h fasted jerboas captured in spring or autumn. Kp10 strongly decreased food intake in fasted female jerboas captured in spring (Fig. 1A) with a significant reduction of food intake as early as 1 hour post-injection (−50%, P < 0.05 compared with vehicle treated animals). In the next 1 h to 5 h post-injection, food intake was inhibited but during the following nocturnal phase, equal values of cumulative food intake were observed 24 h after either peptide or vehicle injections, indicating a late compensatory increase of food intake in the Kp10-treated group. The same dose of Kp10 failed to alter food intake from 1 h up to 24 h post-injection in fasted sexually quiescent females captured in autumn (Fig. 1B). This experiment was repeated once the following year with similar results, confirming a seasonally-dependent anorexigenic effect of Kp10 on food intake in fasted female jerboas. In fasted male jerboas, captured in either spring or autumn, central administration of Kp10 had no significant effect on food intake from 1 h to 24 h post-injection (Fig. 2A,B).

To reveal central sites where Kp10 could act to regulate the female jerboa’s food intake, the level of expression of genes encoding hypothalamic peptides involved in the regulation of food intake was measured 1 h30 post-injection (Fig. 1C). Kp10 injection displayed no effect on the ARC *Npy* mRNA expression in both spring and autumn (P > 0.05). On the other hand, Kp10 injection markedly increased the number of ARC *Pomc* expressing neurons 1 h30 after injection in females in both spring (+57%, P < 0.001) and autumn (+40%, P < 0.01). A two-way ANOVA analysis further revealed a significant season-dependent effect of Kp10 on the number of *Pomc* expressing neurons with a stronger effect in spring than in autumn (F = 41.883; P < 0.001). Furthermore, Kp10 injection induced a season-dependent effect on *Rfrp* gene expression with a decrease in the number of *Rfrp* mRNA expressing neurons at spring (+34% (P < 0.01)) and no significant effect in autumn (P > 0.05). Although female jerboas were fasted for 48 h, it is interesting to note that the number of *Pomc* and *Rfrp* expressing neurons was higher in spring than in autumn in vehicle-injected animals (Fig. 1C, P < 0.05 for *Pomc*, P < 0.001 for *Rfrp*). By contrast, the mean intensity of *Npy* mRNA labeling showed no seasonal variation.

In fasted male jerboas, central injection of Kp10 did not change the number of *Pomc* and *Rfrp* expressing neurons in both spring and autumn but moderately increased *Npy* mRNA expression in spring (p < 0.05) and had no effect in autumn (Fig. 2C). As in females, fasted male jerboas treated with vehicle or Kp10 displayed a higher number of *Pomc* expressing neurons in spring as compared to autumn (F = 19.11, P < 0.01). In contrast, the number of *Rfrp* neurons did not show seasonal variation in fasted control males.

### Effect of central injection of RFRP-3 on food intake and hypothalamic peptide gene expression in ad libitum fed female jerboas at spring or autumn

**Figure 3:**
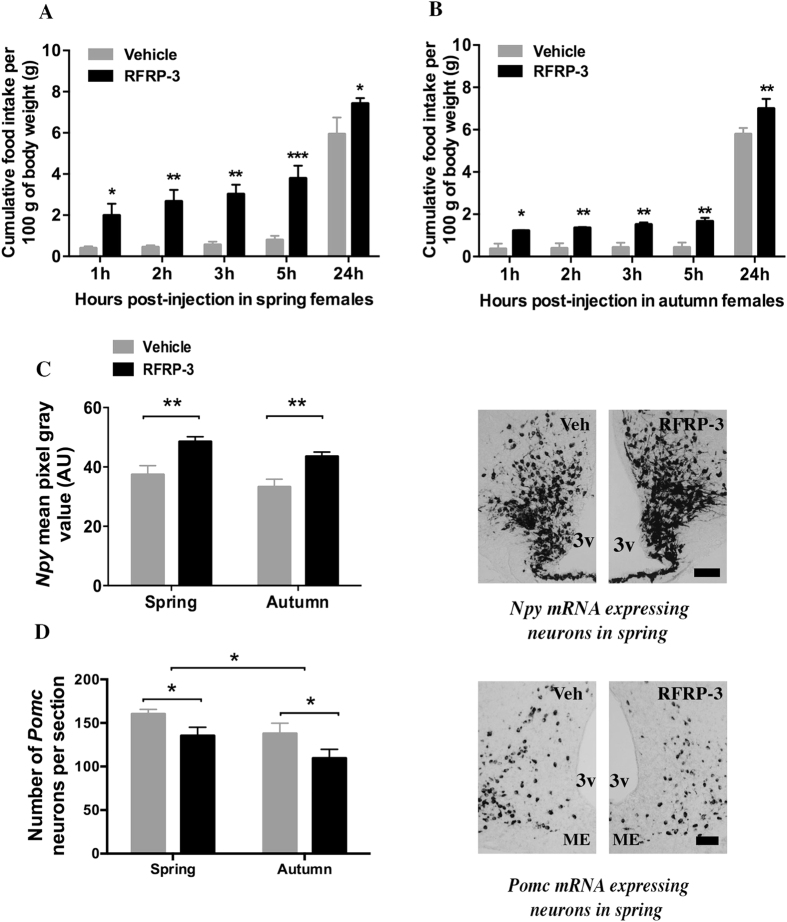
Effect of acute central injection of RFRP-3 on food intake and expression of genes encoding hypothalamic peptides NPY and POMC in ad-libitum fed female jerboas at spring and autumn. (**A,B**) Cumulative food intake (g food intake/100 g body weight) was measured 1 h, 2 h, 3 h, 5 h, and 24 hours following icv injection of 5 μg RFRP-3 or vehicle (0.9% NaCl) in ad-libitum fed female jerboas captured in spring (**A**) or autumn (**B**). Statistical evaluation was performed using two-way repeated measures ANOVA followed by Student-Newman-Keuls test; data are expressed as mean ± SEM (n = 4 per group). ***p < 0.001, **p < 0.01 and *p < 0.05 for significant differences between RFRP-3 and vehicle injected groups; (**C**,**D**) Semi-quantitative analysis of the labeling intensity of *Npy* mRNA in the arcuate nucleus (**C**) and quantitative measure of the number of neurons expressing *Pomc* mRNA in the arcuate nucleus (**D**) 1h30 after 5 μg RFRP-3 (black bars) or vehicle (grey bars) icv injections in spring and autumn female jerboas. Statistical evaluation was performed using two-way ANOVA followed by a post hoc Holm-Sidak test; data are presented as mean ± SEM (n = 4 per group). **p < 0.01 and *p < 0.05 for significant differences between RFRP-3 and vehicle injected group. Representative images of *Npy* (**C**) and *Pomc* (**D**) mRNA labeling in the arcuate nucleus of female jerboas of spring sacrificed 1h30 after RFRP-3 or vehicle icv injections are shown. Scale bars represent 100 μm. AU: arbitrary units; 3v, third ventricle; Veh, vehicle; ME, median eminence.

The effect of central RFRP-3 on food intake was analyzed in females, not male jerboas because only the females were responsive to Kp10 in term of food intake and *Rfrp* gene expression. A single central injection of 5 μg RFRP-3 strongly increased food intake in *ad libitum* fed females in both spring and autumn (Fig. 3A,B). This orexigenic effect of RFRP-3 was strong and significant as early as 1 h post-injection (+395% and +236% for spring and autumn, respectively, in the RFRP-3 treated group as compared to vehicle; P < 0.05) and lasted up to 24 hours post-injection (+25%, P < 0.05, and +21%, P < 0.01, in RFRP-3 compared to vehicle treated animals in spring and autumn respectively). The two-way RM ANOVA revealed a significant effect of treatment (P = 0.003 for spring and P = 0.002 for autumn) and time (P < 0.001 for both seasons), with no dependence of treatment on time. This experiment was repeated once the following year and similar results were obtained, confirming the potent orexigenic effect of RFRP-3 in the female jerboa.

To evaluate the hypothalamic sites of action of RFRP-3, the level of *Npy* and *Pomc* mRNA was measured 1h30 after the injection of peptide or vehicle in spring and autumn female jerboas (Fig. 3C,D). RFRP-3 induced a significant increase in mean ARC *Npy* labeling intensity (+30%, p < 0.01, Fig. 3C) and decrease in the number of ARC *Pomc* expressing neurons (−20%, p < 0.05, Fig. 3D) with no difference between spring and autumn. Notably, in agreement with the previous experiment, the number of *Pomc* expressing neurons is higher in spring as compared to autumn whether jerboas were treated with vehicle or peptide (F = 10.56, P < 0.05).

## Discussion

This study reports that in addition to regulating the reproductive activity, the hypothalamic RF-amide peptides, Kp and RFRP-3, exhibit differential metabolic effects in jerboas. RFRP-3 induces a strong season-independent orexigenic effect in *ad libitum* fed female jerboas while Kp displays a season-dependent anorexigenic effect in female jerboas under negative metabolic state. Furthermore, our data indicate that both peptides may exert their actions via a specific regulation of hypothalamic peptides known to regulate food intake, particularly POMC and NPY.

Recent evidence has pointed the sensitivity of the kisspeptinergic system to negative energy balance states as a decline in the hypothalamic *Kiss1* expression has been reported under conditions of metabolic stress or food restriction in rats and mice^37^,^38^,^39^. Our data show that Kp10 also exerts a rapid and profound anorexinergic effect in female jerboas challenged with 48 h food deprivation. This anorexigenic effect of Kp10 however was observed only in females captured in spring, but not autumn, and was not found when jerboas were fed *ad-libitum*. Of note, when jerboas were food restricted for a shorter time (24 h), Kp10 was still unable to alter food intake (data not shown) probably because these wild semi-desertic rodents are resistant to food deprivation for up to 5 days^40^. In line with these observations, earlier works have reported that Kp10 did not alter food intake in *ad libitum* fed or 12 h-fasted male rats^5^,^37^ or sheep^23^ while a recent study showed that Kp10 reduced food intake in overnight fasted, but not *ad libitum* fed, mice^25^. When Kp10 was injected in male jerboas, whether *ad libitum* fed or fasted, the food intake was similar to vehicle treated animals. Such a sexual dimorphism regarding Kp regulation of metabolism was recently reported in mice^41^. In this study, female KO for the Kp receptor encoding gene (*Kiss1r*) displayed higher body weight and circulating leptin, and impaired glucose tolerance along with lower food intake and energy expenditure, as compared to wild type littermates, while male *Kiss1r* KO mice preserved normal body weight and glucose regulation. Altogether these data support the idea that Kp is anorexigenic under negative energy balance, and in the jerboa this effect appears to be sex- and season-dependent.

In order to further investigate the hypothalamic sites underlying the anorexigenic effect of Kp10 in fasted jerboas, we measured the expression level of genes coding for peptides well known to regulate food intake, NPY and POMC, as well as RFRP-3 because this later peptide displays marked sex-42 and season-16,18,19,31,43 dependent variations in seasonal mammals, including the jerboa. In fasted females, central Kp increased *Pomc* mRNA, with a stronger effect in spring as compared to autumn, which is in agreement with the observed anorexigenic effect of the Kp10 injection. Central Kp also decreased the number of *Rfrp* mRNA expressing neurons in spring, not in autumn. In fasted males, Kp did not alter *Pomc* and *Rfrp* mRNA levels in agreement with the lack of Kp effect on their food intake. Regarding *Npy* gene, Kp displayed a complex regulation with no effect in females and a season-dependent effect in males which might be related to complex sex steroid modulation of Kp action towards NPY neurons^44^.

The anorexigenic effect of Kp observed in fasted spring female jerboas appears to result from an increase in *Pomc* gene expression combined with an inhibition of *Rfrp* gene expression. The Kp effect on jerboa’s POMC neurons is supported by earlier studies reporting in mice that POMC neurons express Kiss1r^29^, are contacted by Kp fibers^28^ and that exogenous Kp activates POMC neurons via a mechanism based on a sodium/calcium exchanger activation and non-selective cation current^29^. However, activation of POMC neurons alone is probably not sufficient to account for the anorexigenic effect of Kp because the increase in *Pomc* mRNA level after Kp injection is observed in both seasons whereas the decrease in food intake only occurs in spring. Remarkably, Kp exhibited a marked season-dependent effect on *Rfrp* mRNA, with an inhibition in spring and no effect in autumn. As far as we know, this is the first report of an inhibitory effect of central Kp on *Rfrp* mRNA, but it is still unclear whether this effect is direct or indirect. A recent study reported that RFRP neurons in male mice do not express Kiss1r and are devoid of Kp fiber projections^45^. However, species or sex differences cannot be excluded since other studies showed that *Kiss1r* mRNA is expressed in the rat DMH^46^ and Kp fiber projections are found in the DMH of female mice^47^. Altogether our data indicate that in fasted female jerboas, central injection of Kp inhibits food intake in spring, but not in autumn, possibly resulting from an increase in *Pomc* gene expression combined with an inhibition of *Rfrp* gene expression.

The *Rfrp* gene encodes different RFRPs among which RFRP-3 regulates reproduction in various mammalian species (for review see refs 2,48). Here, we reveal that RFRP-3 exerts a potent orexigenic effect in the female jerboa as central injection of the peptide induced a marked increase in food intake as early as 1 h and up to 24 h post-injection, with no difference between spring and autumn. We also report that this robust orexigenic effect is associated with an increase in the mRNA level of the orexigenic *Npy* together with a concomitant decrease in the mRNA level of the anorexigenic *Pomc*. Our finding of an orexigenic role of RFRP-3 in the jerboa is in agreement with previous results obtained in rats, mice, sheep and non-human primates^21^,^22^,^23^,^24^, but our data are the first to demonstrate that this metabolic effect might involve an increase in *Npy* and decrease in *Pomc* gene regulation. This finding is supported by earlier reports of the presence of the RFRP receptor and RFRP fiber terminals in the ARC of rodents^8^,^42^,^49^ with some studies showing that RFRP terminals are in close apposition to NPY^30^,^50^ and POMC^30^ neurons. However, studies investigating the effect of RFRP-3/GnIH on POMC and NPY neuronal activity reported paradoxical results since the peptide increased FOS protein in both NPY and POMC neurons in the sheep^23^, inhibited the firing rate of both POMC and NPY neurons in mice^50^ and increased *Npy* but not *Pomc* gene expression in the rat^23^ suggesting species-dependent hypothalamic targets of RFRP-3. In the female jerboa, our study demonstrates that RFRP-3 induces activation of NPY and inhibition of POMC neurons resulting in a robust orexigenic effect. Even though *Rfrp* expression is inhibited by short days in seasonal species, including the jerboa^14^,^16^,^19^,^31^, and RFRP receptor expression is reduced in the ARC of short-day adapted hamsters^42^, we observed no difference in the effect of RFRP-3 on food intake in spring compared to autumn.

**Figure 4:**
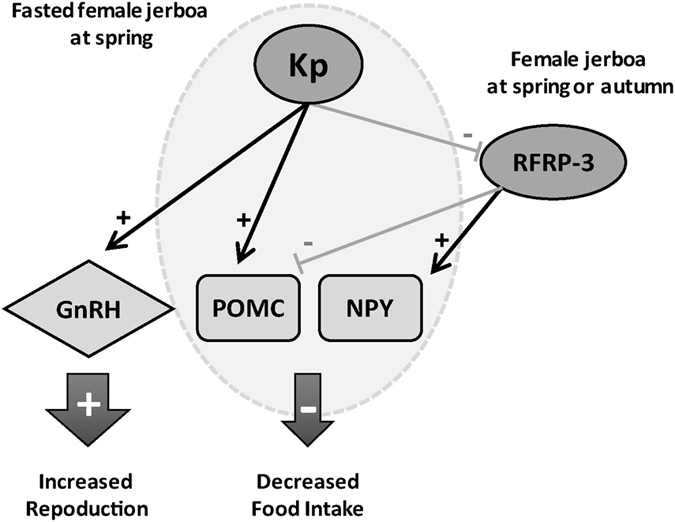
Working model showing a hypothalamic pathway used by kisspeptin and RFRP-3 to regulate food intake and reproduction in the female jerboa. In spring when female jerboas are under negative energetic balance, elevated level of kisspeptin (Kp) stimulates the reproductive axis by activating GnRH neurons and inhibits food intake via activation of POMC neurons and inhibition of RFRP-3 neurons, the latter exerting an increase in orexigenic *Npy* and a decrease in anorexigenic *Pomc*. Of note, the orexigenic effect of RFRP-3 is found independent of season.

In conclusion, our study demonstrates a sexually dimorphic and season-dependent anorexigenic effect of Kp in jerboas under negative metabolic state possibly via a combined increase in *Pomc* and decrease in *Rfrp* gene expression. The differential effect of Kp on females’ food intake may be related to their different sexual and energy status between both seasons. Indeed at spring, when coming out of hibernation, female jerboas are sexually active with reduced body weight^35^ and express higher ARC Kp immunoreactivity^18^ as compared to autumn. It might be interesting to compare Kiss1r distribution and Kp neuronal connection to POMC and RFRP neurons between spring and autumn to help understand the seasonal differences in the metabolic action of Kp. The observation that Kp injection increases *Pomc* mRNA in females in both spring and autumn is in agreement with previous electrophysiological studies in mice which shows that POMC neurons are directly regulated by Kp^29^. Furthermore, our findings that Kp inhibits *Rfrp* mRNA in spring, point to RFRP neurons as a target through which Kp transmits its season-dependent inhibition of food intake. Indeed, our data show a marked orexigenic effect of RFRP-3 via a combined increase in *Npy* and decrease in *Pomc* gene expression observed in both spring and autumn even in *ad libitum* fed female jerboas. Therefore, one might hypothesize that when female jerboas are submitted to strong reproductive and metabolic challenge in spring, Kp increases reproduction via the activation of GnRH neurons and reduces food intake by activating POMC and inhibiting RFRP neurons (Fig. 4).

It has been reported that Kp and RFRP-3 exhibit opposite stimulatory and inhibitory effects, respectively, on female rodent reproduction^8^,^12^,^47^,^51^,^52^ and whether this is also true in female jerboas should be investigated. Additionally, this study reveals that both peptides also display opposite anorexigenic, induced by Kp, and orexigenic, induced by RFRP-3, effects through the recruitment of different metabolic hypothalamic peptides. These findings strengthen our hypothesis that reproductive activity and food intake are coordinated in wild jerboas^31^, and here we provide a hypothalamic model including differential roles of Kp and RFRP-3 for the understanding of synchronized seasonal regulation of reproduction and energy balance.

## Methods

### Animals

Male and female sexually mature jerboas (*Jaculus orientalis*), weighing 100–160 g (n = 94) were captured from the Atlas Mountains of Morocco (altitude 1565 m) in spring (May) when they were sexually active, and autumn (October) when their reproductive activity was shut down. The captured animals were transported to the animal facility at the University of Fes where they were allowed to adapt to captivity for one week. Sexual maturity was estimated based on the morphological parameters of their sexual organs. After acclimatization, animals were put in individual cages under natural conditions of temperature and photoperiod with *ad libitum* access to food (vegetables, sunflower and barley seeds). At the University of Fes, where the experiments were performed, experimental protocols do not require approval by an institutional and/or licensing committee. However, the experimental protocols had been approved by a French ethics committee for a previous study on a similar animal model, the Syrian hamster. Further, all experiments were conducted in accordance with the international guidelines for the Care and Use of Mammals in Neurosciences and Behavioral Research (2003).

### Intracerebroventricular cannulation

Intracerebroventricular (icv) cannula implantation was performed according to a protocol previously established in rodents^13^. Animals were anesthetized with an intraperitoneal injection of Imalgene 500 (ketamine 50 mg/ml) and Rompun (xylazine 20 mg/kg) and positioned in a stereotaxic frame. An incision was made in the scalp along the midline of the animal’s head, and a hole was drilled through the skull over the implantation coordinates. Then a stainless steel 22-gauge cannula (Plastics One, Roanoke, VA, USA) was stereotaxically implanted in the lateral ventricle (stereotaxic coordinates were 2 mm lateral to the midline, 0.6 mm posterior to the Bregma and 3.5 mm inferior to the dura mater) and fixed to the skull by bone screws and dental cement. The cannula was sealed with a metallic wire protected with a plastic cap. After surgery, the animals were put back in their individual cages and checked for proper recovery for one week with free access to food before peptide injection.

### Peptide administration

Mouse Kp10 (**YNWNSFGLRY-NH2**, MW: 1300 g) was synthetized by GenScript (Piscataway, NJ, USA) and Jerboa RFRP-3 (peptide sequence deduced from *Jaculus orientalis Rfrp* gene sequence^31^: **ILSPIPNLPGRF-NH2**, MW: 1323 g) was synthetized by CASLO (CASLO Laboratory ApS, Lyngby, Denmark). Due to limitation in the number of jerboas captured in the wild, we could not perform a proper dose-response curve to test the peptide effects. Therefore, the doses of the peptide to be injected were first chosen according to the doses reported to be used in various rodent species, including ours done in Syrian hamster^13^. The dose chosen for Kp (4 μg in 5 μl 0.9% NaCl) was validated by measuring another biological parameter, the production of testosterone, well known to be increased by an efficient dose of Kp. For RFRP-3, we tested 2 doses (1.5 μg and 5 μg in 5 μl 0.9% NaCl) and only 5 μg/μl was sufficient to alter food intake. Injections were carried out in the early light phase (8 h–11 h). The animals were submitted to a light gaseous anesthesia, then 5 μl of peptide solution or vehicle (0.9% NaCl) were injected during 5 minutes using a 28-gauge stainless steel cannula attached to polyethylene tubing and a 5 μl Hamilton syringe (Hamilton Inc., Reno, NV). After recovery from anesthesia, each jerboa was placed in an individual cage and challenged for food intake or brain peptide gene expression.

### Experimental designs

#### Effect of central injection of Kp10 or RFRP-3 on food intake

In a first set of experiments, central injection of 4 μg Kp10 was tested in *ad libitum* fed male and female jerboas and was found to display no effect on food intake (data not shown). This dose however induced an expected increase in testosterone production in spring male jerboas sacrificed 1h30 post-injection (from 1.78 ± 0.61 nmol/L, in 4 vehicle injected animals to 6.46 ± 1.35 nmol/L in 4 Kp10 injected animals, p < 0.01). As it has been reported that Kp10 alters food intake in fasted mice^25^, male and female jerboas were fasted for 48 h before Kp10 or vehicle injections in either spring (May/June) or autumn (October/November) with 4 animals per experimental group (n = 4). In a second set of experiments, the effect of central injection of either 1.5 μg or 5 μg RFRP-3 or vehicle was tested on *ad libitum* fed female jerboas either in spring or autumn with 4 animals per experimental group, and only the group injected with 5 μg RFRP-3/μl showed an altered food intake.

In both experiments, jerboas were put back in their individual home cages with a preweighed amount of food immediately after icv injection. Food intake was thereafter measured 1 h, 2 h, 3 h, 5 h and 24 h post-injection.

#### Effect of central injection of Kp10 or RFRP-3 on hypothalamic peptide gene expression

To identify the putative central sites of Kp10 or RFRP-3 action for their metabolic effects, expression of genes encoding hypothalamic peptides involved in food intake and/or metabolic regulation was analyzed after the peptide injections. Kp10 (4 μg) or vehicle was injected in 48 h fasted male and female jerboas, in either spring or autumn (n = 4/group, except for the autumn female group with n = 3) and RFRP-3 (5 μg) or vehicle was injected in *ad libitum* fed female jerboas in either spring or autumn (n = 4/group). The central injections were performed as described above, except that 1h30 post-injection, animals were deeply anesthetized with an intraperitoneal injection of ethyl urethane (1 ml/100 g, Acros Organics) and were transcardially perfused with 50 ml isotonic saline solution (NaCl 0.9%) followed by 250 ml of a fixative solution containing 4% paraformaldehyde (PFA, Sigma-Aldrich) in 0.1 M phosphate buffer (PB) at pH 7.6. In male spring jerboas, blood was taken by cardiac puncture for further testosterone measurement. In order to avoid early mechanical stimulation of neuronal or glial cells when taking out skull bones, animal’s skulls (devoid of skin, eyes, ears and neck) were first kept in 4% PAF for 1 h, then the brains were removed from the skull, post-fixed at 4 °C in the same fixative solution for 24 h, dehydrated in ethanol baths of increasing concentrations and stored in butanol until embedded in polyethylene glycol (PEG, Acros Organics) as described previously^31^,^53^. Serial 12 μm microtome sections were cut throughout the hypothalamus using a Leica microtome, mounted on Superfrost^®^ ultraplus slides under RNAse free conditions and stored at −80 °C until processed for *in situ* hybridization.

### *In situ* hybridization

Non-radioactive *in situ* hybridization for *Npy, Pomc* and *Rfrp* mRNA was performed according to a protocol previously validated^31^. Briefly, sense and antisense riboprobes containing a 87–522 rat *Npy* (Genbank NM_012614.2), a 157–731 rat *Pomc* (Genbank NM_139326), and a 614 bp *Jaculus orientalis Rfrp*, were transcribed from linearized plasmids in the presence of digoxigenin-labeled nucleotides according to the manufacturer’s protocol (Dig RNA Labelling Kit, Roche Diagnostics, Mannheim, Germany). For each animal, one brain section every 144 μm was selected throughout the rostral, middle and caudal ARC for *Npy* and *Pomc*, and DMH/VMH for *Rfrp*. Sections of all peptide and vehicle treated spring and autumn animals were treated together under identical conditions. Brain sections were post-fixed in 4% PAF for 10 min, treated with 0.5 μg/ml proteinase K (Roche, Meylan, France) for 30 min at 37 °C, and acetylated twice in triethanolamine buffer for 10 min. Hybridization was performed for 40 hours at 60 °C with 200 ng/ml labeled antisense probes in 50% formamide, 5X SSC, 5X Denhardt’s solution, 0.1% Tween20 and 1 mg/mL salmon sperm DNA. Six stringency rinses were performed at 72 °C with 0.1X SSC and 0.05% Tween20 for 10 min each. Digoxigenin-labeled bound probes were detected with an alkaline phosphatase-labeled anti-digoxigenin antibody (1/5000, Roche, Meylan, France). After detection, the slides were mounted using Crystal mount aqueous mounting medium (Sigma-Aldrich, Lyon, France) and coverslipped with Eukitt (Sigma-Aldrich, Lyon, France).

### Cell counting and semi-quantitative analysis

In a previous study^31^, we reported that quantification of RFRP, POMC and NPY encoding gene expression in the jerboa’s hypothalamus gave similar seasonal variation when analyzing the number of labeled neurons or the intensity of labeling per neuron. Therefore for quantification of *Pomc* and *Rfrp* mRNA expression, labeled neurons were manually counted on a Leica DMRB microscope (Leica Microsystems). For each animal, *Pomc* and *Rfrp* expressing neurons were counted on both sides of the ventricle throughout the rostro-caudal levels of ARC for *Pomc* and DMH/VMH for *Rfrp*. For each experimental condition, the value given is the number of labeled neurons per section ± SEM counted in the 4 animals/group. Due to the high density and close proximity of NPY neurons in the ARC, a semi-quantitative analysis of the *Npy* labeling intensity was performed as described previously^31^. Gray scale (256 levels) tiff images were taken at 10X magnification at 2776 × 2074 pixel using a Leica DMRB microscope (Leica Microsystems) with an Olympus DP50 digital camera (Olympus France). For analysis of ARC *Npy* expression, three sections were taken to represent rostral, middle, and caudal regions of the ARC. For each animal, a rostral, middle and caudal ARC section was photographed on both sides of the ventricle and for each slide a background image without a brain section was measured and subtracted from the sample images. The labeled area of NPY neurons distribution was delineated and the mean pixel gray value of the *Npy* mRNA labeling was determined using Image J software (NIH Image, Bethesda MD, USA). For each animal, the 3 measured mean pixel gray values from the 3 sections were used to calculate the mean pixel gray value per animal. These individual values were then used to calculate the mean ± SEM intensity of *Npy* mRNA staining for each experimental condition (n = 4 for each group).

### Statistical analysis

All data are given as mean ± SEM. Significance of peptide versus vehicle injection on food intake was analyzed by two-way repeated measures ANOVA followed by Student-Newman-Keuls test when appropriate. Difference in the level of neuropeptide gene expression between vehicle- and peptide-injected groups in the two seasons was analyzed using two-way ANOVA followed by post hoc Holm-Sidak test. The threshold for statistical significance was set at p < 0.05. All analyses were performed using SigmaPlot version 12.5 (Systat Software Inc., San Jose, CA, USA) and all graphs were designed using GraphPad Prism version 6 (GraphPad software Inc., San Diego, CA, USA).

## Additional Information

**How to cite this article**: Talbi, R. *et al*. Kisspeptin and RFRP-3 differentially regulate food intake and metabolic neuropeptides in the female desert jerboa. *Sci. Rep*. **6**, 36057; doi: 10.1038/srep36057 (2016).

**Publisher’s note**: Springer Nature remains neutral with regard to jurisdictional claims in published maps and institutional affiliations.
